# Neuropsychological characteristics of adults with attention‐deficit/hyperactivity disorder without intellectual disability

**DOI:** 10.1002/npr2.12134

**Published:** 2020-08-30

**Authors:** Masami Hida, Wakaho Hayashi, Yuka Okajima, Osamu Takashio, Akira Iwanami

**Affiliations:** ^1^ Department of Psychiatry Showa University School of Medicine Tokyo Japan; ^2^ Department of Psychiatry and Neurology Showa University East Hospital Tokyo Japan; ^3^ Showa University Karasuyama Hospital Tokyo Japan

**Keywords:** adult, attention‐deficit disorder with hyperactivity, intelligence, memory

## Abstract

**Aims:**

While several studies have reported various cognitive impairments in children with attention‐deficit/hyperactivity disorder, the neuropsychological profiles of adults with this disorder are understudied. Here, the intelligence and memory functions of adults with attention‐deficit/hyperactivity disorder without intellectual disability were evaluated.

**Methods:**

The Wechsler Adult Intelligence Scale—Third Edition and Wechsler Memory Scale—Revised were administered to 30 adults with attention‐deficit/hyperactivity disorder whose full‐scale intelligence quotients were >85. Diagnoses were based on the Diagnostic and Statistical Manual of Mental Disorders‐IV criteria. Conners' Adult ADHD Rating Scales—Self‐Report—screening version and the Autism Spectrum Quotient were also evaluated.

**Results:**

In the Wechsler Adult Intelligence Scale—Third Edition, the verbal intelligence quotient was significantly higher than the performance intelligence quotient and the verbal comprehension score was the highest among the secondary indices. In the Wechsler Memory Scale—Revised, the visual memory score was the highest measure. Although the verbal intelligence quotient had no correlation with any Wechsler Memory Scale—Revised measures, the performance intelligence quotient was significantly correlated with the visual memory and attention scores of the Wechsler Memory Scale—Revised. Conners' Adult ADHD Rating Scales hyperactive‐impulsive score was significantly correlated with the verbal intelligence quotient, whereas the inattention score was not correlated with any measures of the Wechsler Adult Intelligence Scale—Third Edition or Wechsler Memory Scale—Revised.

**Conclusions:**

The results suggest that while adults with normal‐intelligence attention‐deficit/hyperactivity disorder have comparatively high verbal comprehension and social knowledge, their ability of information processing and visual‐motor coordination are relatively weak.

## Abstract 2

The Wechsler Adult Intelligence Scale—Third Edition (WAIS‐III) and Wechsler Memory Scale—Revised (WMS‐R) of 30 adults with attention‐deficit/hyperactivity disorder (ADHD) were examined. The verbal intelligence quotient was significantly higher than the performance intelligence quotient in the WAIS‐III and the performance intelligence quotient was significantly correlated with the visual memory and attention scores of the WMS‐R. Adults with ADHD have comparatively high verbal comprehension and social knowledge, while their information processing ability and visual‐motor coordination are relatively weak.

## Graphical abstract

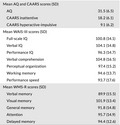

## 1 INTRODUCTION

Attention‐deficit/hyperactivity disorder (ADHD) is characterized by inattention, hyperactivity, and impulsivity. While it was formerly believed that childhood ADHD recedes in adulthood, recent research has found that ADHD symptoms persist well into adulthood.^1^ Biedermann et al^2^ reported that in the process of transition from childhood to adulthood, the improvement in inattention symptoms was markedly limited compared to that in hyperactivity and impulsivity. In recent years, the number of individuals who first receive a diagnosis of ADHD in adulthood has increased, and ADHD in adulthood is garnering attention.

Previous studies have indicated that children with ADHD show significant dysfunction in a wide range of neuropsychological domains, including executive function.^3^ The findings of the Wechsler Intelligence Scale for Children reporting that the verbal intelligence quotient (VIQ) is higher than the performance intelligence quotient (PIQ) in children with ADHD are relatively robust. Conversely, the neuropsychological characteristics of adults with ADHD have not been intensively investigated. Notably, considerable numbers of adults with ADHD receive medical treatment while they remain socially well‐adjusted and continue to be successful in their work life. It is crucial to examine the neuropsychological profiles of the adult population in relation to their symptoms.

Thus, in this study, we investigated the intelligence, memory function, and symptoms of adults with ADHD without intellectual disability. The Wechsler Adult Intelligence Scale—Third Edition (WAIS‐III), a standard psychological test for evaluating intelligence,^4^ and the Wechsler Memory Scale—Revised (WMS‐R), a frequently used measurement to assess several memory dimensions,^5^ were simultaneously used for the first time. As the clinical symptoms and impairments in ADHD often persist from childhood into adulthood, we predicted that the intellectual characteristics of adult ADHD would resemble those of childhood ADHD. Additionally, we hypothesized that memory function would tend to be low overall due to the symptoms of ADHD.

## 2 METHODS

We administered the WAIS‐III and WMS‐R to 30 adults with ADHD (17 males and 13 females). The subjects were recruited at the outpatient clinic of Showa University East Hospital or Showa University Karasuyama Hospital. Only those with an ADHD diagnosis according to the Diagnostic and Statistical Manual of Mental Disorders (DSM)‐IV criteria and Conners' Adult ADHD Diagnostic Interview for DSM‐IV™^6^, ^7^ were included. Participants with intellectual disability (full‐scale IQ < 85) or with severe physical disorders, any other psychiatric disorder, or substance abuse or dependence were excluded. The Japanese version of Conners' Adult ADHD Rating Scales (CAARS)—Self‐Report—screening version (CAARS‐SR‐SV) and Japanese version of the Autism Spectrum Quotient (AQ) were also assessed^8^, ^9^, ^10^ in order to evaluate the participants' subjective symptoms. The participants' ages ranged from 18 to 59 with a mean of 31.4 years (standard deviation; SD: 10.8), and the average duration of education was 14.3 years (SD: 1.9; ranged from 12 to 18 years). The CAARS average inattentive (IA) subscale score was 18.2 (SD: 6.1; ranged from 6 to 27) and the hyperactive/impulsive (HI) subscale score was 9.1 (SD: 6.2; ranged from 1 to 23), which were higher than those in healthy adults.^8^ The average AQ score was 31.5 (SD: 6.5; ranged from 15 to 41), which was also higher than that of the healthy adults, although it was lower than the cutoff of 33 points for autism spectrum disorder (ASD).^10^

SPSS 22.0J (IBM Corp.) was used for all statistical analyses. The means with standard deviations were calculated for the WAIS‐III, WMS‐R, AQ, and CAARS. The paired t test was used to calculate the VIQ‐PIQ difference of the WAIS‐III. One‐way repeated‐measures analysis of variance was used to compare the secondary indices of the WAIS‐III and each measurement of the WMS‐R; post hoc pairwise comparisons were performed with Bonferroni correction. Pearson's product‐moment correlation coefficients were calculated among the measurements of the WAIS‐III and WMS‐R as well as among the measurements of clinical symptoms and the WAIS‐III and WMS‐R. The significance level was set at 0.05, except for the correlations, at which 0.01 was set to count for the possible type I error.

## 3 RESULTS

Table 1 shows the mean AQ, CAARS, WAIS‐III, and WMS‐R scores of the participants. All measurements of the WAIS‐III were within the normal range. VIQ was significantly higher than PIQ (*t*(29) = 2.855, *P* < .01). Concerning the secondary indices, significant within‐participants effects were found (*F*(2.42) = 4.723, *P* < .01, ηp^2^ = 0.14) with post hoc pairwise comparisons, revealing that the score of verbal comprehension (VC) was significantly higher than that of working memory (WM; *P* < .05). All measurements of the WMS‐R were also within the normal range. The within‐participants effects were also significant (*F*(2.21) = 6.654, *P* < .01, ηp^2^ = 0.187) with the pairwise comparison resulting in the score of visual memory being significantly higher than that of verbal memory (*P* < .01), general memory (*P* < .01), and delayed memory (*P* < .05).

**TABLE 1:** Mean scores of the AQ, CAARS, WAIS‐III, and WMS‐R

Mean AQ and CAARS scores (SD)	
AQ	31.5 (6.5)
CAARS inattentive	18.2 (6.1)
CAARS hyperactive‐impulsive	9.1 (6.2)
Mean WAIS‐III scores (SD)	
Full‐scale IQ	100.8 (14.1)
Verbal IQ	104.1 (14.8)
Performance IQ	96.3 (14.7)
Verbal comprehension	104.8 (16.5)
Perceptual organization	97.4 (15.2)
Working memory	94.6 (13.7)
Performance speed	93.7 (17.6)
Mean WMS‐R scores (SD)	
Verbal memory	89.9 (15.5)
Visual memory	101.9 (13.4)
General memory	91.8 (14.8)
Attention	95.7 (14.9)
Delayed memory	94.4 (12.6)

Abbreviations: AQ, Autism Quotient; CAARS, Conners' Adult ADHD Rating Scale; IQ, intelligence quotient; SD, standard deviation; WAIS‐III, Wechsler Adult Intelligence Scale—Third Edition; WMS‐R, Wechsler Memory Scale—Revised.

Pearson's product‐moment correlation coefficients were calculated between the IQ values of the WAIS‐III and WMS‐R (Table 2). VIQ had no correlation with the WMS‐R, while PIQ showed a significant correlation with visual memory (*r*(29) = 0.606, *P* < .001) and attention (*r*(29) = 0.588, *P* < .01). The full‐scale intelligence quotient (FIQ) only had a significant correlation with attention (*r*(29) = 0.512, *P* < .01).

**TABLE 2:** Correlation coefficients between WAIS‐III and WMS‐R

WMS‐R	WAIS‐III						
	VIQ	PIQ	FIQ	VC	PO	WM	PS
Verbal memory	0.330	0.117	0.275	0.256	0.274	0.283	−0.067
Visual memory	0.111	0.606**	0.383	−0.087	0.640**	0.528*	0.376
General memory	0.317	0.268	0.343	0.200	0.420	0.393	0.048
Attention	0.323	0.588*	0.512*	0.106	0.661**	0.659**	0.191
Delayed memory	0.419	0.222	0.390	0.332	0.343	0.395	−0.089

Abbreviations: FIQ, full‐scale intelligence quotient; PIQ, performance intelligence quotient; PO, perceptual organization; PS, performance speed; VC, verbal comprehension; VIQ, verbal intelligence quotient; WAIS‐III, Wechsler Adult Intelligence Scale—Third Edition; WM, working memory; WMS‐R, Wechsler Memory Scale—Revised.

* *P* < .01.

** *P* < .001.

Table 3 shows the Pearson product‐moment correlation coefficients between measurements of clinical symptoms and WAIS‐III and WMS‐R scores. While AQ and the CAARS‐IA score showed no significant correlation with any of the measures, the CAARS HI score was significantly correlated with VIQ (*r*(29) = −0.466, *P* < .01).

**TABLE 3:** Correlation coefficients between WAIS‐III, WMS‐R, and clinical symptoms

	WAIS‐III						
	VIQ	PIQ	FIQ	VC	PO	WM	PS
AQ	−0.150	−0.012	−0.104	−0.182	0.046	0.080	−0.013
CAARS Inattentive	−0.431	−0.121	−0.347	−0.346	−0.118	−0.187	0.011
CAARS hyperactive‐impulsive	−0.466*	−0.245	−0.431	−0.377	−0.264	−0.320	0.032

	WMS‐R				
	Verbal memory	Visual memory	General memory	Attention	Delayed memory
AQ	−0.023	−0.005	−0.023	0.081	−0.110
CAARS inattentive	−0.367	−0.113	−0.355	−0.129	−0.395
CAARS hyperactive‐impulsive	−0.313	−0.090	−0.308	−0.182	−0.327

Abbreviations: AQ, Autism Quotient; CAARS, Conners' Adult ADHD Rating Scales; FIQ, full‐scale intelligence quotient; PIQ, performance intelligence quotient; PO, perceptual organization; PS, performance speed; VIQ, verbal intelligence quotient; VC, verbal comprehension; WAIS‐III, Wechsler Adult Intelligence Scale—Third Edition; WM, working memory; WMS‐R, Wechsler Memory Scale—Revised.

* *P* < .01

** *P* < .001.

## 4 DISCUSSION

To our knowledge, this is the first study to simultaneously examine the profiles of the WAIS‐III and WMS‐R in adults with ADHD. Although the intelligence level of the participants in the present study was within the normal range, VIQ was significantly higher than PIQ. This result is consistent with previous findings in children and adults with ADHD^11^, ^12^ and suggests that while individuals with ADHD have comparatively high verbal comprehension and social knowledge, their ability of information processing and coordination of visual‐motor functions are relatively weak. The finding of higher VIQ compared to PIQ in this study may be associated with the relatively high AQ score in the participants compared with neurotypicals^10^, although none were diagnosed with ASD. With several studies reporting higher VIQ in individuals with ASD,^11^, ^12^ a similar neuropsychological impairment may underlie both ADHD and ASD.

The mean score of visual memory was highest among the measurements of the WMS‐R, although the memory function of the participants was within the normal range, as was their WAIS‐III scores. Relatively lower verbal, general, and delayed memory may be caused by the symptoms of ADHD, as these three scores showed negative, although nonsignificant, correlations with the CAARS‐IA score. Moreover, these results correspond to the difficulties in daily life individuals with ADHD often face, such as trouble concentrating on tasks and being forgetful of completing tasks. The heterogeneity or imbalance of functions reflected in the scores of the WAIS‐III and WMS‐R may affect the daily and social life of individuals with ADHD.

For the CAARS scores, the IA subscale score was higher than the HI subscale score, indicating the predominance of inattention symptoms. This matches with previous findings that inattention symptoms largely remain in adulthood^2^ and shows that our sample was fairly typical of adult ADHD.

Regarding the association between clinical symptoms and neuropsychological measurements, verbal function impairment was most closely related to ADHD symptoms as the CAARS HI score showed negative correlations with VIQ. Previous neurophysiological and neuroimaging studies have indicated decreased prefrontal function in individuals with ADHD,^13^ which may correspond to these results.

Contrary to our expectation, the WMS‐R measurements of the participants were within the normal range. However, as Quinlan et al^14^ reported, compared to healthy adults, a high proportion of adults with ADHD showed a large dissociation between VIQ on the WAIS and logical memory (a subitem of verbal memory) on the WMS, which may explain our results. The logical memory test of WMS‐R is a task in which the tester reads aloud two stories to a participant, and after both stories, the participant recalls and retells the stories. Active information processing is necessary to process and summarize the story while listening and to further recall it, which can be extremely difficult for individuals with ADHD for whom auditory information processing and simultaneous processing are compromised. Another subscale of verbal memory is known as verbal paired associates, in which a tester verbally presents pairs of words, after which, the tester presents just one word from the pair, and the participant recalls and responds with the other word of the pair. This task also requires auditory information retention and recall, and, as expected, is considered to be difficult for individuals with ADHD. In contrast, VIQ includes numbers of items assessing crystallized ability such as comprehension, knowledge, and vocabulary, as well as task presentation both in visual and in auditory form, rendering them easier for individuals with ADHD to handle.

Several limitations of our study must be considered. First, only 30 participants were included, limiting the statistical power of the study. Second, we did not include normal controls as comparison. Third, possible effects of medication were not considered. At the time of our assessment, 10 of the 30 participants were on either methylphenidate or atomoxetine. Fourth, only subjective measurements of self‐rating scales were used to assess symptoms, lacking objective clinician rating scales.

This study highlights the uneven intelligence and memory profiles of adults with ADHD with possible relation to symptoms. Further studies that determine the association of daily functioning and medication regimens with objective measures of symptoms are to be planned.

## CONFLICT OF INTEREST

The authors declare no conflict of interest.

## AUTHOR CONTRIBUTIONS

MH was critically involved in the analysis of the data and wrote the first draft of the manuscript. WH contributed to the interpretation of the data and the writing of the manuscript. AI was critically involved in the study design and analysis of the data and contributed to the interpretation of the data and the writing of the manuscript. YO and OT were involved in the subject recruitment process and the clinical diagnostic assessments. All authors contributed to and approved the final manuscript.

## APPROVAL OF THE RESEARCH PROTOCOL BY AN INSTITUTIONAL REVIEWER BOARD

The study protocol has been approved by the suitably constituted Research Ethical Committee of the Showa University School of Medicine (No. 793), and it conforms to the provisions of the Declaration of Helsinki.

## INFORMED CONSENT

All participants provided written consent to the study after a full explanation of the study procedures.

## Data Availability

Research data are not shared. This is because the participants did not consent for open data sharing.
